# Novel polygenic risk score associates with diverticulitis in a multi-institutional, ancestrally diverse cohort

**DOI:** 10.1038/s41598-025-26455-6

**Published:** 2025-11-27

**Authors:** Christopher J. Neylan, Thomas Ueland, Sarah A. Abramowitz, John S. DePaolo, Noah L. Tsao, Jeffrey L. Roberson, Alexander T. Hawkins, David J. Carey, Diane T. Smelser, Michael G. Levin, Rebecca L. Hoffman, Scott M. Damrauer, Lillias H. Maguire

**Affiliations:** 1 Department of Surgery, Perelman School of Medicine, University of Pennsylvania, Philadelphia, PA 19104 USA; 2 Vanderbilt University School of Medicine, Nashville, TN 37232 USA; 3 The Donald and Barbara Zucker School of Medicine at Hofstra/Northwell, Hempstead, NY 11549 USA; 4 Division of General Surgery, Section of Colon & Rectal Surgery, Vanderbilt University Medical Center, Nashville, TN 37232 USA; 5 Department of Genomic Health, Weis Center for Research, Geisinger Health System, Danville, PA USA; 6 Cardiovascular Institute, Department of Medicine, Perelman School of Medicine, University of Pennsylvania, Philadelphia, PA 19104 USA; 7 Corporal Michael Crescenz VA Medical Center, Philadelphia, PA 19104 USA; 8 Division of Colorectal Surgery, Geisinger Medical Center, Geisinger Commonwealth School of Medicine, Danville, PA USA; 9 Department of Genetics, Perelman School of Medicine, University of Pennsylvania, Philadelphia, PA 19104 USA; 10 Division of Colon and Rectal Surgery, Department of Surgery, Perelman School of Medicine, University of Pennsylvania, Philadelphia, PA 19104 USA; 11 Division of Colorectal Surgery, Hospital of the University of Pennsylvania, 3400 Spruce Street, 4 Silverstein, Philadelphia, PA 19104 USA

**Keywords:** Diseases, Gastroenterology, Genetics, Medical research, Risk factors

## Abstract

Diverticulitis is a common and morbid colorectal disease that recurs after an initial attack in up to 30% of patients. Elective surgery to remove the affected portion of the colon is an effective intervention to prevent disease recurrence, but appropriate patient selection is challenging given the limited ability to predict which patients are likely to recur, or the severity with which they may do so. Genetics influence diverticulitis and can be used for risk stratification, but this has only been studied in European ancestry populations. Using state-of-the-art techniques, we created a polygenic risk score that associates with diverticulitis prevalence and severity and is externally validated on meta-analysis in a diverse patient population across three different biobanks. This work represents the first published diverticulitis polygenic score to demonstrate utility in associating specifically with diverticulitis in non-European populations, and may provide the basis for clinical implementation in elective surgical decision-making.

## Introduction

Diverticulitis is a significant health problem in the United States and it recurs after the initial episode in up to 30% of patients^1–3^. Elective resection of the diseased segment of the colon is the only effective preventive measure, but the inability to reliably predict recurrence, or the severity thereof, makes it challenging to decide which patients would benefit from surgery. ^4–10^ This uncertainty is reflected in formal guidance from professional societies, which leaves the decision up to the individual surgeon and patient rather than providing uniform criteria for assessment^5,6^. Improved risk stratification is necessary in diverticulitis to inform surgical decision-making and patient counseling. One potential strategy is using individual genetic data codified in a polygenic risk score.

Twin studies have suggested that 40–53% of the risk of diverticulitis is heritable, and subsequent genome-wide association studies (GWASs) for diverticular disease have identified numerous potentially implicated single nucleotide polymorphisms (SNPs)^11–17^. A polygenic risk score (PRS) is a sum of the small risk contributions of individual SNPs,^18–21^ and evidence suggests that this cumulative measure can powerfully predict disease risk even when individual SNPs have minimal predictive potential^22–25^. The application of PRSs to diverticulitis offers the potential to improve the prediction of recurrent and severe diverticulitis. Previously published PRSs for diverticulitis have been limited by performance degradation in non-European ancestry participants or by the inability to discriminate diverticulitis from diverticulosis^14,26,27^. Indeed, limited cross-population applicability is a well-documented problem in the genomics literature^28,29^.

The aim of the current study is to create a novel PRS for diverticulitis based on summary data from the largest GWAS meta-analysis for diverticular disease, and subsequently to determine whether the PRS associates with prevalent and/or severe diverticulitis in ancestrally diverse populations across three different biobanks as a form of external validation^30,31^.

## Methods

### Overview of workflow

**Fig. 1:**
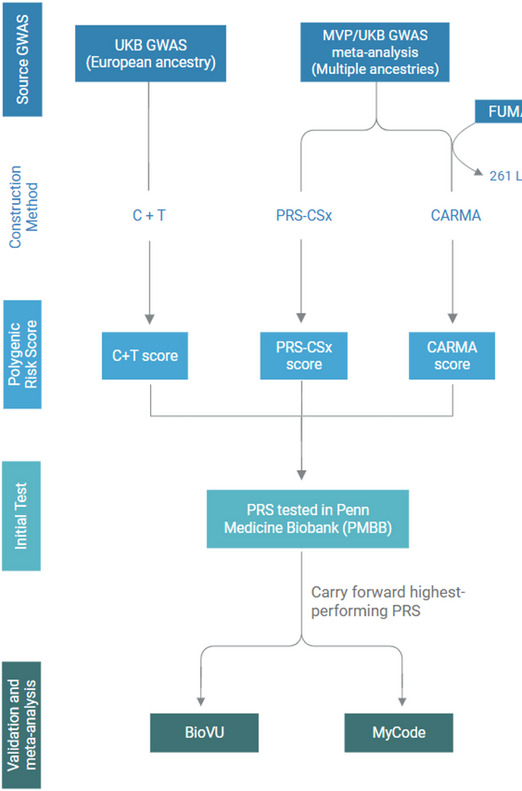
Overview of study workflow demonstrating the creation of multiple polygenic risk scores (PRSs) and initial testing in the PMBB, followed by validation in BioVu and MyCode, with meta-analysis performed among the three cohorts. UKB = United Kingdom Biobank, MVP = MillionVeteran Program, FUMA = Functional Mapping and Annotation of Genome-Wide Association Studies.

This study can be schematically broken down into three steps (Fig. 1). First, we created three polygenic risk scores using three different PRS construction methods: clumping and thresholding (C + T), CARMA, and PRS-CSx. Next, we tested the three PRSs in the Penn Medicine Biobank (PMBB) and determined the best-performing score. Finally, we carried forward the best-performing score for external validation testing in the BioVu and MyCode biobanks, and meta-analyzed the results across all three biobanks. All methods were performed in accordance with relevant guidelines and regulations. Specifically, polygenic risk scores were created and assessed in accordance with expert consensus guidelines for PRS reporting and predictive modeling,^19,32,33^ and this research was conducted in accordance with federal regulations protecting human subjects research (45 CFR 46.104).

### Target populations

The Penn Medicine Biobank (PMBB) consists of participants enrolled by the University of Pennsylvania Healthcare System. There are at least 247,342 participants in the PMBB of whom at least 43,982 have undergone genome-wide genotyping and whole exome sequencing^34^. The MyCode Community Initiative consists of at least 170,503 individuals recruited from the Geisinger Health System, and has been described in detail elsewhere^35^. BioVu is the Vanderbilt University Medical Center biobank and includes approximately 325,000 participants, with cohort details given elsewhere^36^.

### Cohort creation and phenotype definitions

The following phenotypic classes were defined: severe diverticulitis (complicated diverticulitis requiring a drainage/surgical procedure), non-severe diverticulitis (diverticulitis not meeting severe criteria), and diverticulosis only (i.e. diverticulosis without diverticulitis). Prevalent or total diverticulitis refers to diverticulitis of any kind (either severe or non-severe). Slightly different phenotypic criteria were utilized for each biobank to reflect the unique set of variables and coding structures contained therein. In all cases, the criteria were validated via manual chart review. As an illustration, complete phenotyping details utilized in the PMBB can be found in Supplemental Table 1.

**Table 1:** Combined cohort characteristics.

Characteristic	Total	PRS Deciles 1-9^2^	Top PRS Decile	p-value^3^
Sample Size				
N	36077	32471	3606	
Diverticulosis only				
Yes	29694 (82.3%)	27076 (83.4%)	2618 (72.6%)	<0.001
Non-severe Diverticulitis				
Yes	5306 (14.7%)	4534 (14.0%)	772 (21.4%)	<0.001
Severe Diverticulitis				
Yes	1077 (3.0%)	861 (2.7%)	216 (6.0%)	<0.001
Diverticulitis (Total)				
Yes	6383 (17.7%)	5395 (16.6%)	988 (27.4%)	<0.001
Sex				
Male	16861 (46.7%)	15241 (46.9%)	1620 (44.9%)	.022
Age, years				
Mean (SD)	70.0 (± 12.0)	70.2 (± 12.0)	67.9 (+12.6)	<0.001
Ancestry				
African	3017 (8.4%)	2633 (8.1%)	384 (10.6%)	<0.001
European	32638 (90.5%)	29456 (90.7%)	3182 (88.2%)	
Other	422 (1.2%)	382 (1.2%)	40 (1.1%)	<0.001
Body Mass Index				
Mean (SD)	31.0 (± 6.7)	30.9 (+ 6.7)	31.4 (± 6.9)	<0.001
Missing	33 (0.1%)	32 (0.1%)	1 (0.0%)	.250
Smoker				
Yes	19321 (53.6%)	17334 (53.4%)	1987 (55.1%)	0.51
Missing	1 (0.0%)	1 (0.0%)	0 (0.0%)	1.00
Type II Diabetes Mellitus				
Yes	15467 (42.9%)	13866 (42.7%)	1601 (44.4%)	.051

1 Cohort combines PMBB, BioVU, and MyCode data.

2 PRS: Polygenic Risk Score.

3 P-values calculated using t-tests for continuous variables and Fisher’s exact tests for categorical variables.

Testing and validation of the PRSs were performed in a cohort consisting only of individuals with diverticular disease (i.e. prevalent diverticulitis or diverticulosis). The goal in defining diverticular disease as an inclusion criterion was to ensure that any association between diverticulitis and the PRS was discovered relative to those with diverticulosis, thereby mitigating the possibility that the PRS would detect an association with diverticulosis rather than diverticulitis. Individuals with Crohn’s disease, ulcerative colitis, colon cancer, or ischemic colitis were excluded, as were those less than 18 years of age at enrollment in the respective biobank. Population cohorts (e.g. European or African) were defined using genetically inferred ancestry based on genetic principle component similarity to the 1000 genomes reference population^34,37^. In accordance with the recommendations of the National Academies of Science, Engineering, and Medicine, we have attempted to avoid the use of racial descriptors^38^.

### Polygenic risk score pipeline

#### PRS creation

Three PRSs for diverticulitis were included in this analysis.

##### C + T PRS

The C + T PRS was previously defined based on a GWAS of diverticular disease in the European-ancestry cohort of the United Kingdom Biobank (UKB)^13,27,39^. Using this PRS, we calculated scores in the PMBB using PLINK (v1.90) in R^20^.

##### CARMA PRS

CARMA is a method of statistical fine-mapping that identifies putatively causal variants within pre-defined loci in GWAS summary statistics, and assigns variants weights based on summary statistic effect sizes^40^. Based on a recent GWAS meta-analysis of a multi-ancestry Million Veteran Program (MVP) and UKB combined cohort,^31^ we used FUMA (*r*^*2*^ < 0.01, window size 500 kb) to identify 261 risk loci^41^. The Million Veteran Program is a genomic database that includes a relatively high proportion of African population participants^42^. We then employed CARMA to find independently significant SNPs within each locus, discovering a total of 1,787 variants and producing a corresponding polygenic weight file with 1,787 weights ranging in absolute value from 0.008 to 0.157. PLINK was then used to calculate CARMA PRSs in the PMBB.

##### PRS-CSx PRS

PRS-CSx is a Bayesian statistical method that calculates a weight for every SNP included in the genotyping array^43^. Instead of relying on pre-defined loci, PRS-CSx infers posterior effect sizes and applies a continuous shrinkage model, which has the advantage of improving cross-ancestry PRS applicability by leveraging linkage disequilibrium across populations^43^. The weight file produced by PRS-CSx included 1,270,605 variants, with weights ranging in absolute value from 6.92 _X_ 10^− 11^ to 0.0197 (see Supplemental Data for the full PRS-CSx weights file), based on the same GWAS summary statistics utilized for the CARMA PRS^31^. In order to capture the full cross-ancestry transferability of PRS-CSx, we utilized pgsc_calc (specifically the znorm2 scaling feature) to calculate PRS-CSx scores in the PMBB^44^.

#### PRS testing in the PMBB

After deriving the three PRSs described above, each was tested in the PMBB for association with diverticulitis and severe diverticulitis. Statistical analyses were performed in R (v 4.2.0, R Foundation for Statistical Computing, Vienna, Austria). Population characteristics were compared (Supplemental Table 2) and prevalence plots were generated in the overall cohort as well as the European and African populations (Supplemental Fig. 1) to estimate the prevalence of diverticulitis and severe diverticulitis in each PRS decile. Univariable analyses were conducted using Fisher’s exact test (Supplemental Fig. 2) to ascertain whether high polygenic risk (top risk decile) was associated with increased odds of diverticulitis compared to the remainder of the population, and whether low polygenic risk (bottom decile) was associated with decreased odds of diverticulitis. This was repeated for severe diverticulitis. A multivariable logistic regression model (Supplemental Fig. 3) was constructed to determine the odds of having prevalent diverticulitis based on polygenic risk (treated as a continuous variable) while controlling for age, sex, smoking, BMI, and type two diabetes mellitus (T2DM). This was repeated for severe diverticulitis. Both the univariable and multivariable analyses were run in three populations (overall, European, and African) for all three PRSs (data in supplemental figures is shown for the best-performing score (PRS-CSx)). Subpopulation analyses were limited to the European and African populations as these were the only cohorts sufficiently powered for subgroup analysis.

Finally, the performance of each PRS was assessed^32,33^. The performance attributable to each PRS was determined by computing the area under the receiver operating curve (AUC) for a regression model including PRS, age, sex, and the first five genetic principal components, and comparing this to the AUC for the same regression model lacking PRS. This analysis was run for models of prevalent diverticulitis and severe diverticulitis, and was repeated in subgroups of European and African population participants in the PMBB. We then ran additional analyses to compare the AUC of a model consisting of: (1) PRS alone, (2) age, sex, and BMI, (3) PRS + age, sex, and BMI. We did this in the PMBB for both diverticulitis and severe diverticulitis.

### Absolute risk estimate

In order to provide measures of absolute risk, we calculated the absolute risk of diverticulitis and, separately, of severe diverticulitis, in our PMBB cohort, stratified by PRS quartile. Absolute risk was defined as the number of individuals with the outcome divided by the total number of individuals at risk. In order to provide an estimate of lifetime risk, we assumed a mean age of 70 years (the mean age in our cohort) and calculated annual risk as absolute risk divided by the observation period, which was assumed to begin at age 45 (the current recommended age for colon cancer screening), and finally we presumed a life expectancy of 80 years to calculate a lifetime risk of diverticulitis and severe diverticulitis for the individual’s remaining lifespan.

### External validation and meta-analysis

**Fig. 2:**
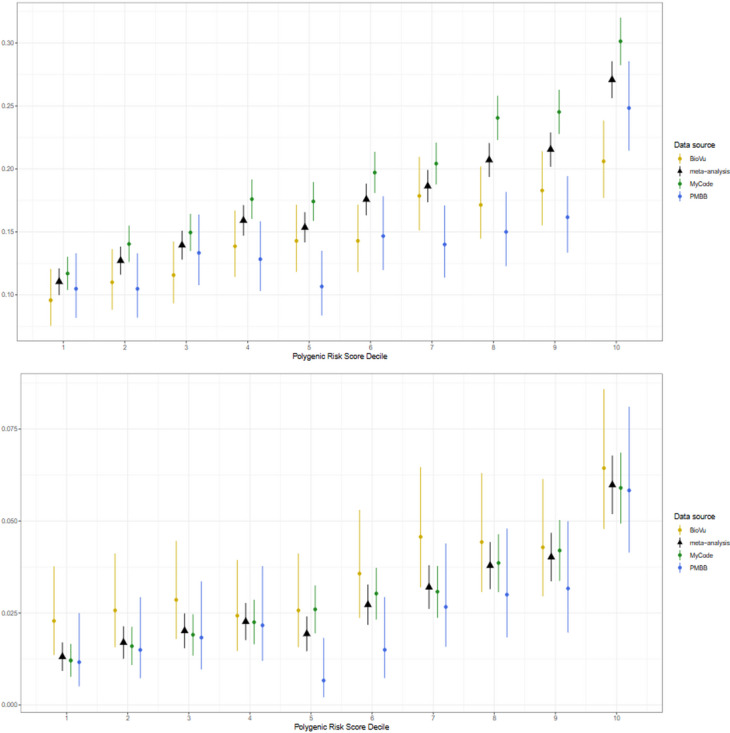
Prevalence plots of (**a**), total (severe and non-severe) diverticulitis and (**b**), severe diverticulitis using a fixed-effects, inverse-variance weighted meta-analysis across cohorts from the Penn Medicine Biobank (PMBB), BioVU, and MyCode.

**Fig. 3:**
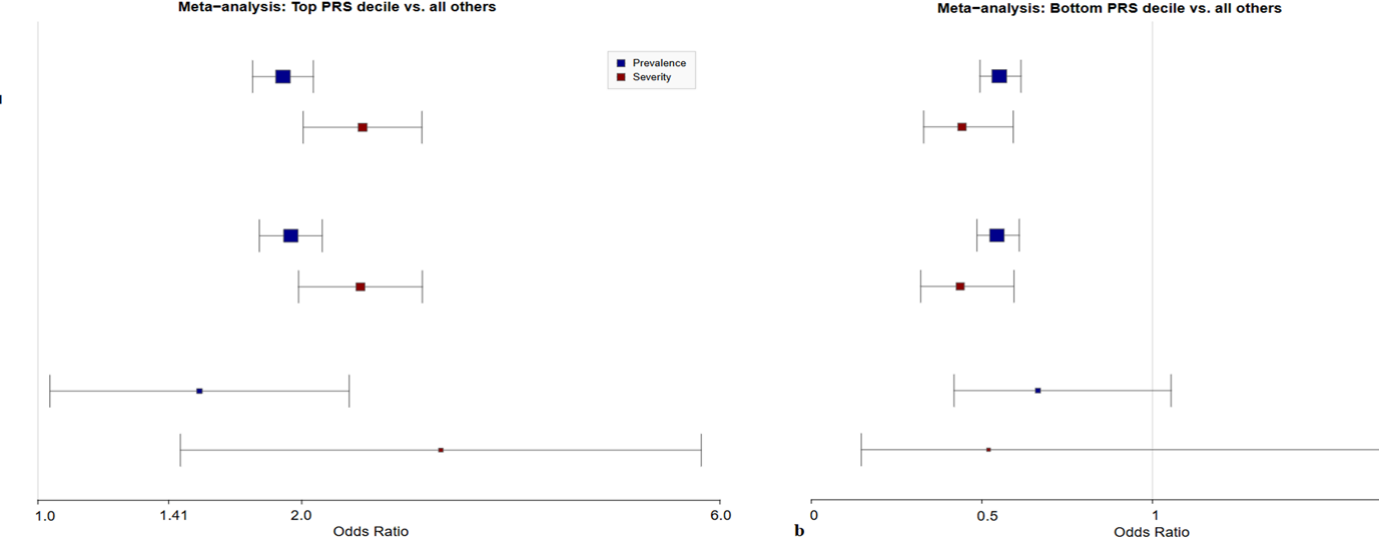
Univariable analysis forest plot. Meta-analyzed odds of prevalent (severe and non-severe) and severe diverticulitis on univariable analysis for a, the top polygenic risk decile compared to the remaining 90% of the cohort, and b, the bottom polygenic risk decile compared to the remaining 90% of the cohort. Whiskers denote 95% confidence interval. Larger odds ratio boxes signify smaller p-values.

**Fig. 4:**
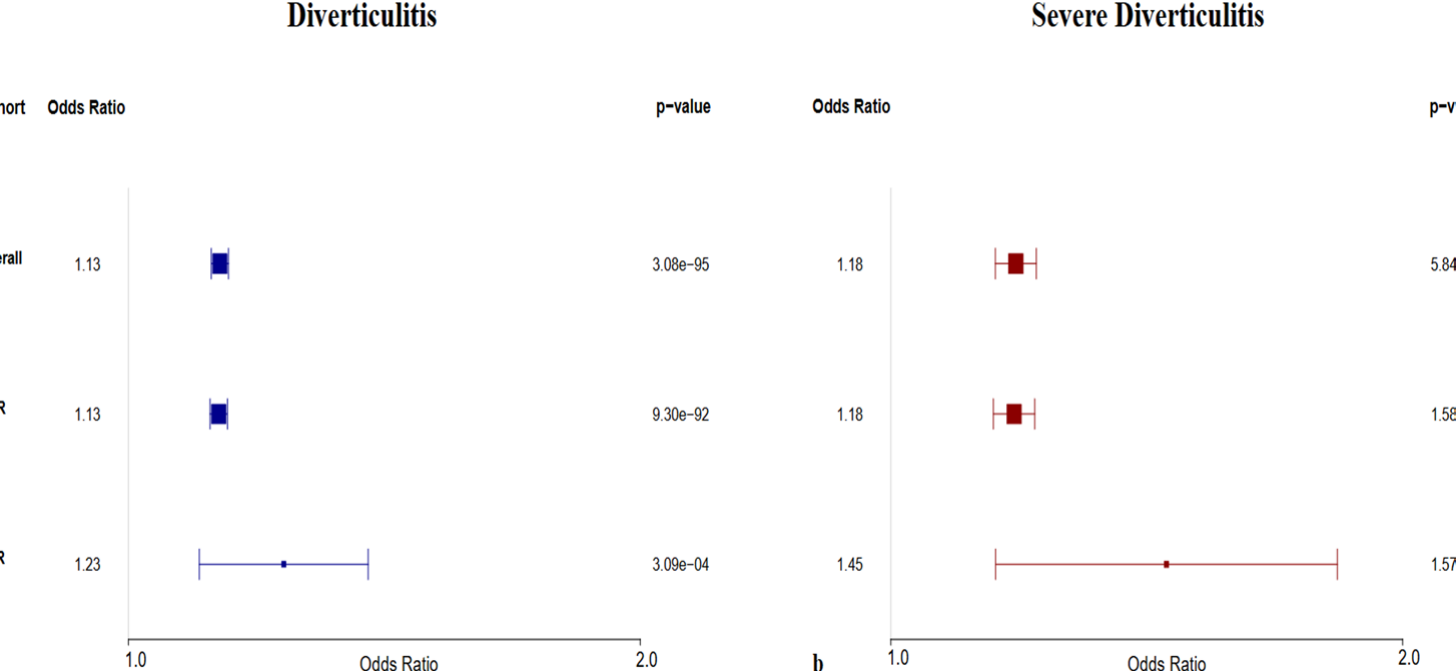
Multivariable regression. Meta-analyzed odds of (**a**), total (severe and non-severe) diverticulitis, and (**b**), severe diverticulitis, on multivariable logistic regression controlling for several clinical co-variates. Whiskers denote 95% confidence internval. Larger odds ratio boxes signify smaller p-values.

**Fig. 5:**
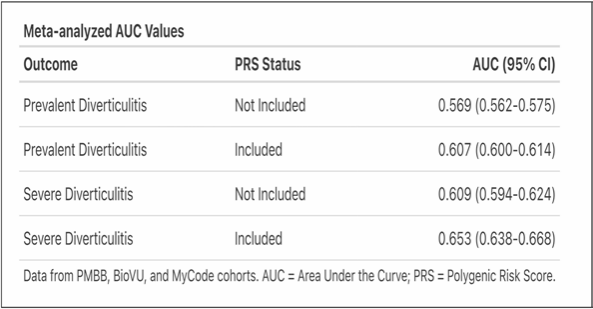
Meta-analyzed area under the curve (AUC) analysis showing the PRS-attributable increase in AUC for models of prevalent (severe and non-severe) and severe diverticulitis.

The best-performing PRS in the PMBB was defined as the score with the greatest PRS-attributable AUC increase. The weight file for this score was shared with collaborators at BioVU and MyCode, who then calculated PRSs in those biobanks using pgsc_calc. The PRS was tested in each biobank using the same testing methods employed in the PMBB. Summary data from each biobank was shared and cohort characteristics were pooled and analyzed (Table 1). An inverse-variance weighted, fixed-effects meta-analysis was performed to generate meta-analyzed prevalence plots for diverticulitis and severe diverticulitis (Fig. 2), meta-analyzed univariable analyses mirroring those performed in the PMBB (Fig. 3), meta-analyzed multivariable regressions mirroring those performed in the PMBB (Fig. 4), and meta-analyzed AUC analysis (Fig. 5). The three biobanks were assumed to be independent given their geographical distance from each other.

## Results

### Cohort overview

The final meta-analyzed cohort consisted of 36,077 individuals with diverticulosis or diverticulitis, of whom 6,383 (17.7%) had prevalent diverticulitis (inclusive of severe and non-severe diverticulitis) and 1,077 (3.0%) had severe diverticulitis (Table 1). When stratified into high (top decile) and low (deciles one through nine) polygenic risk on the basis of PRS-CSx score, the baseline rates of clinical characteristics such as age, sex, and BMI were similar between the two groups, but the rates of diverticulitis and severe diverticulitis were significantly higher in the high polygenic risk group. Complete descriptive statistics of the meta-analyzed cohort are provided in Table 1.

### Prevalence plots

There was a clear upward trend in the prevalence of diverticulitis and severe diverticulitis as polygenic risk increased. This was particularly true at the top risk decile. On meta-analysis, the prevalence of diverticulitis in the top risk decile was 27%, an approximate 2.5-fold increase from the 11% prevalence in the bottom decile. Similarly, the rate of severe diverticulitis in the top decile was 6.0%, a more than 4-fold increase from the 1.3% rate in the bottom decile. The plots for meta-analyzed prevalent and severe diverticulitis in the overall cohort are displayed in Fig. 2. PMBB-specific prevalence plots in the overall, EUR, and AFR populations are displayed in Supplemental Fig. 1.

### Univariable analysis

The results displayed in Fig. 3 demonstrate, on meta-analysis, significantly increased odds of prevalent (OR 1.90, 95% CI 1.75–2.06) and severe (OR 2.35, 95%CI 2.00–2.74) diverticulitis among participants in the top polygenic risk decile compared to the remainder of the cohort. These relationships persisted in the subgroups of European population (prevalence OR 1.94, 95% CI 1.79–2.11; severity OR 2.33, 95% CI 2.00–2.75) and African population (prevalence OR 1.53, 95% CI 1.03–2.27; severity OR 2.88, 95% CI 1.45–5.71) individuals. In the overall cohort, the bottom decile of polygenic risk was significantly associated with decreased odds of prevalent (OR 0.55, 95% CI 0.49–0.61) diverticulitis, and while this trend was true for severe diverticulitis and in the ancestry-specific subpopulations, the result was variably significant (overall cohort severity: OR 0.44, 95% CI 0.32–0.59) (in the European-ancestry cohort, prevalence OR 0.54, 95% CI 0.48–0.61 and severity OR 0.44, 95% CI 0.32–0.59; in the African-ancestry cohort, prevalence OR 0.66, 95% CI 0.41–1.05 and severity OR 0.52, 95% CI 0.15–1.85). PMBB-specific univariable analyses for PRS-CSx in the overall, EUR, and AFR populations are given in Supplemental Fig. 2. Univariable analyses in the PMBB for the CARMA PRS were similar to PRS-CSx in terms of significance and direction of results, but with slightly attenuated ORs; the C + T PRS did not achieve consistently significant associations with prevalent and severe diverticulitis across cohorts (data not shown).

### Multivariable analyses

The results displayed in Fig. 4 reveal that an increase in polygenic risk is significantly associated with increased odds of prevalent and severe diverticulitis on meta-analysis, even when controlling for clinically relevant confounders. Furthermore, it shows that these effects persist in subpopulation analyses limited to participants genetically similar to the African reference population. Given the lack of consistently significant association between the bottom decile of polygenic risk and decreased odds of diverticulitis on univariable analysis, multivariable models were not constructed for this outcome.

PMBB-specific multivariable regression modeling results are given in Supplemental Fig. 3, with full details including the performance of all variables included in the model provided in Supplemental Fig. 4. The CARMA PRS achieved similar results though, as on univariable analyses, with slightly attenuated ORs (data not shown). The C + T PRS did not achieve consistently significant associations with prevalent and severe diverticulitis across cohorts (data not shown).

### Proof-of-concept analyses

In an attempt to test the performance a simpler PRS with fewer variants, the five SNPs with the largest effect sizes were individually tested to see if the presence of the effect allele associated with prevalent or severe diverticulitis on univariable analyses, and four of the five were significantly associated with prevalent diverticulitis while the fifth SNP was significantly associated with severe diverticulitis. A PRS consisting of only these five SNPs was significantly associated with prevalent diverticulitis on multivariable logistic regression, however it failed to improve the AUC and it was not associated with severe diverticulitis (data not shown).

### Absolute risk

We estimated the absolute risk of diverticulitis in our cohort stratified by PRS quartile, and found that in the first (lowest) PRS quartile, the absolute risk of diverticulitis was 0.109 (95% CI 0.094–0.126); in the second PRS quartile was 0.122 (0.106–0.140); in the third quartile was 0.143 (0.125–0.161); and in the highest PRS quartile was 0.196 (0.176–0.217). For severe diverticulitis, in the first (lowest) PRS quartile, the absolute risk was 0.014 (95% CI 0.009–0.021); in the second PRS quartile it was 0.015 (0.010–0.023); in the third quartile it was 0.022 (0.016–0.031); and in the highest PRS quartile it was 0.043 (0.033–0.054). Under the assumptions described in the methods we then calculated an absolute lifetime risk of diverticulitis in the first (lowest) PRS quartile of 0.043 (95% CI 0.037–0.049); in the second PRS quartile of 0.048 (0.042–0.054); in the third PRS quartile of 0.056 (0.049–0.063); and in the highest PRS quartile of 0.076 (0.068–0.083). Similarly, the absolute lifetime risk of severe diverticulitis in the first (lowest) PRS quartile was 0.006 (95% CI 0.003–0.008); in the second PRS quartile was 0.006 (0.004–0.009); in the third PRS quartile was 0.009 (0.006–0.012); and in the highest PRS quartile was 0.017 (0.013–0.021).

### Model fitness

To ascertain the PRS-attributable increase in AUC, diverticulitis and severe diverticulitis were modeled using age, sex, and genetic principal components with and without the inclusion of the PRS. PRS-CSx was the best-fitting model by AUC in the PMBB (severe diverticulitis AUC increase from 0.62 [95% CI 0.60– 0.65] to 0.66 [95% CI 0.64–0.69]; diverticulitis AUC increase from 0.57 [95% CI 0.56– 0.589] to 0.61 [95% CI 0.593–0.62]). The population-specific subgroup analysis revealed a PRS-attributable AUC increase for models of prevalent diverticulitis in both European (from 0.55 [95% CI 0.54–0.56] without PRS to 0.59 [0.57–0.60] with PRS) and African (from 0.52 [0.49–0.54] to 0.57 [0.54–0.60]) population subgroups, and a PRS-attributable AUC increase in models of severe diverticulitis (European 0.60 [0.56–0.64] to 0.65 [0.61–0.69] and African 0.66 [0.60–0.73] to 0.71 [0.64–0.78], though this did not always reach statistical significance particularly in severe diverticulitis, perhaps owing to decreased sample size. The additional AUC analyses revealed that for the outcome of diverticulitis, a model consisting of PRS alone had an AUC of 0.576 (95% CI 0.564–0.589); a model of age, sex, and BMI had an AUC of 0.521 (0.502–0.54), and a model of these variables combined had an AUC of 0.572 (0.559–0.585). For severe diverticulitis, a model consisting of PRS alone had an AUC of 0.630 (0.600–0.660); a model of age, sex and BMI had an AUC of 0.622 (0.598–0.646); and a model of these variables combined had an AUC of 0.659 (0.633–0.685).

The meta-analyzed AUC increases across the three biobanks are given in Fig. 5, which demonstrate a significant increase in the AUC from 0.61 (95% CI 0.59–0.62) to 0.65 (95% CI 0.64–0.67) for severe diverticulitis, and a smaller but still significant AUC increase for prevalent diverticulitis.

## Discussion

This paper describes the creation and testing of several polygenic risk scores (PRSs) for diverticulitis in the Penn Medicine Biobank (PMBB), the most successful of which (PRS-CSx) was externally validated in two biobanks (Vanderbilt’s BioVU and Geisinger’s MyCode). On meta-analysis across the PMBB, BioVU, and MyCode, a high PRS was associated with significantly increased odds of prevalent and severe diverticulitis — a finding which remained true exclusively among African population participants, making this the first diverticulitis PRS to function well in a non-European population subgroup.

Those in the highest decile of polygenic risk were more than twice as likely to have severe diverticulitis, and almost twice as likely to have diverticulitis, as other participants (Fig. 3). These results persisted in subpopulation analyses of European-ancestry and African-ancestry individuals, although the PRS generally performed slightly better in European populations than in African populations (Fig. 3). On multivariable regression controlling for potentially confounding clinical factors, an increased PRS was significantly associated with increased odds of prevalent and severe diverticulitis, and this again remained true in European and African subpopulations (Fig. 4). Notably, all analyses took place in a cohort of patients who all had diverticulosis, thus mitigating the possibility that the PRS associates with diverticulosis and instead suggesting that it associates with diverticulitis per se. It should be noted that the PRS was more successful in associating with high risk of disease rather than low risk of disease. This constrains the use of the PRS to high-risk individuals, but we believe these is the most clinically relevant identification because it may help identify those for whom the risk of surgery is justified and also identify those likely to benefit from lifestyle interventions is important to optimize resources.

The success of this PRS represents an important advancement in the diverticulitis polygenic risk scoring literature^14,26,27^. One of the main research challenges in diverticular disease is to disentangle diverticulosis from diverticulitis, and this is the only PRS to function well in non-European populations and not suffer from confounding between diverticulosis and diverticulitis, as recent diverticular disease PRSs have^14,45^. Moreover, area under the curve (AUC) analyses are not comparable between our PRS and other recently published PRSs given that the other PRSs were tested in part for association with diverticulosis, rather than tested only for association with the more clinically relevant diverticulitis, as ours was^14,45^.

This study has high translational potential. One potential area of clinical relevance is informing surgical decision-making. There remains substantial clinical equipoise in the decision to recommend elective surgery for recurrent diverticulitis. Although surgeons are comfortable performing risk stratification in extreme clinical scenarios, such as a young patient with multiply recurrent diverticulitis or an elderly patient with a single episode of mild disease, decision-making is less certain in more nuanced circumstances. This is evidenced by the high variability in rates of elective colectomy, ambiguous societal guidelines, and a recent randomized trial of sigmoidectomy versus non-operative management^5,6,46^. Additionally, a previous study of 213 colorectal surgeons revealed high utility of a genetic risk tool in indeterminate clinical scenarios^27^. Unlike proposed endoscopic, radiologic, or clinical risk stratification tools, PRS is objective and does not suffer from reverse causality^7–10^. In such cases, a PRS may help push the proverbial needle toward or away from preventative surgery.

A second potential clinical use of our PRS, is to help motivate lifestyle change. We believe, for example, that informing diverticulitis patients with a high PRS that their genetic risk for diverticulitis is elevated, we might be able—with careful counseling that emphasizes the role of environmental effects on disease development—to convey the importance of lifestyle (e.g. diet and exercise) changes to overcome the genetic predisposition.

Our study should be interpreted in the context of its limitations. First, diverticulitis it not a purely genetic disease. Environmental factors, like diabetes and smoking, may play at least as much of a role in the development of disease as do genetics, and so this PRS cannot be a standalone prognostic tool. This limitation is reflected in the modest PRS-attributable AUC improvement. Next, the lack of environmental data (e.g. dietary information, immunosuppressive use) precluded us from controlling for these factors on multivariable analysis, which may bias the data in unpredictable ways, though may be mitigated by the inclusion of BMI, diabetes, and smoking, which can correlate with a generally healthy lifestyle. Additionally, the PRS-CSx score requires imputation, which is more expensive and potentially harder to scale than a simpler PRS inclusive of fewer SNPs. An area of future research should be to develop a PRS that maintains predictive power but is cheaper to implement and more computationally efficient. Finally, although this study includes patients of diverse ancestries, the cohort is largely European and African-ancestry derived. Individuals of other ancestries are represented in relatively small numbers in these biobanks, therefore we did not perform subpopulation analyses in these groups; additionally, in certain analyzed subgroups such as African-population participants with a high PRS and severe diverticulitis, the numbers of included participants are relatively small. This leads us to advocate for reproduction in larger databases, however we still believe the current findings contribute to the existing literature. In particular Biobank Japan, Sapien (India), and emerging biobanks in Africa offer opportunities for future research and comparison, though many of the represented countries have a lower incidence of diverticular disease.

Our group recently published a critique of coronary heart disease polygenic risk scores, demonstrating that scores with similar population-level performance produced inconsistent individual-level risk estimates^47^. This critique, in a recent *JAMA* paper, pertains to phenotypes for which several polygenic scores exist with similar population-level performances and the choice of which to carry forward for prospective validation is unclear. In contrast, the diverticulitis polygenic risk score presented herein is the only diverticulitis score to perform well in non-European populations and not be confounded by association with diverticulosis, and thus it stands out as the preferred score in which to invest the time and resources for prospective validation.

## Conclusion

Our findings provide evidence that a polygenic score created utilizing the summary statistics of a large, multi-ancestry genome-wide association study can associate with prevalent and severe diverticulitis on meta-analysis of a multi-institutional, diverse patient population. The robustness of this association in the African population represents an advancement beyond previously-published diverticulitis risk scores, and raises the possibility that such a score can be incorporated in an equitable fashion into the clinical care of diverticulitis patients who, based on clinical data alone, do not have a clear answer as to whether their future risk of diverticulitis is high enough to merit preventative surgery.

## Supplementary Information

Below is the link to the electronic supplementary material.

Supplementary Material 1

Supplementary Material 2

Supplementary Material 3

Supplementary Material 4

Supplementary Material 5

Supplementary Material 6

## Data Availability

All computational code used for analysis will be made available upon reasonable request. Please direct requests for data to Dr. Lillias Maguire via email at Lillias.Maguire@Pennmedicine.upenn.edu. Summary data on which the GWAS meta-analysis was performed is publicly available. Please see the source GWAS meta-analysis of the United Kingdom Biobank and Million Veteran Program (https://docs.google.com/spreadsheets/d/1AeeADtT0U1AukliiNyiVzVRdLYPkTbruQSk38DeutU8/edit#gid=903887429). Penn Medicine Biobank, BioVu, and MyCode all abide by strict privacy and confidentiality standards in order to protect the health information of their participants. None of these databases are publicly available. For each biobank, informed consent for collection and use of their data was obtained from each participant prior to enrollment (further details about these biobanks including their informed consent processes can be found at the following links: for PMBB, https://pmbb.med.upenn.edu/; for Biovu, https://victr.vumc.org/what-is-biovu/; for Mycode, https://www.geisinger.edu/gchs/research/mycode)
