# Supplementary material for: Novel polygenic risk score associates with diverticulitis in a multi-institutional, ancestrally diverse cohort
Source: Sci Rep. 2025 Nov 27;15:42348. doi: 10.1038/s41598-025-26455-6 (PMC12660771; doi:10.1038/s41598-025-26455-6)
Supplement: Supplementary file 3 — Supplementary Material 3 [file 41598_2025_26455_MOESM3_ESM.docx]

**Supplemental Table 1. PMBB Phenotype definitions**

| *Diagnosis* | *ICD-10* or CPT** codes* |
| --- | --- |
| Diverticulosis only | At least **one** encounter with and ICD-10 code below AND does not meet criteria for Prevalent diverticulitis  ICD-10: K57.30, K57.31, K57.50, K57.51, K57.90, K57.9, |
| Uncomplicated diverticulitis | At least **two** encounters with an ICD-10 code below AND does not meet criteria for Severe diverticulitis  ICD-10: K57.20, K57.21, K57.32, K57.33, K57.40, K57.41, K57.52, K57.53, K57.80, K57.81, K57.92, K57.93 |
| Severe diverticulitis | At least **one** encounter with an Uncomplicated diverticulitis ICD-10 code **AND** percutaneous drainage or surgery (defined by any of the CPT codes below) during an emergency/inpatient encounter  CPT: 75989, 49060, 49405, 49406, 49407, 49020, 49322, 49323, 44140, 44141, 44143, 44144, 44145, 44146, 44150, 44151, 44157, 44160, 44188, 44320, 44204, 44205, 44206, 44207, 44208, 44210, or 44212 |
| Prevalent (or all) diverticulitis | Meets criteria for either Uncomplicated diverticulitis or Severe diverticulitis |
| Ulcerative colitis^†^ | ICD-10: K51.00, K51.011, K51.012, K51.014, K51.018, K51.019, K51.20, K51.211, K51.213, K51.218, K51.219, K51.30, K51.311, K51.319, K51.40, K51.412, K51.419, K51.50, K51.511, K51.518, K51.519, K51.80, K51.811, K51.813, K51.814, K51.818, K51.819, K51.90, K51.911, K51.912, K51.913, K51.914, K51.918, K51.919 |
| Colon cancer^†^ | ICD-10: C18.0, C18.1, C18.2, C18.3, C18.4, C18.6, C18.7, C18.8, C18.9, C19, C20 |
| Crohn’s disease^†^ | ICD-10: K50.00, K50.011, K50.012, K50.013, K50.014, K50.018, K50.019, K50.10, K50.111, K50.112, K50.113, K50.114, K50.118, K50.119, K50.80, K50.811, K50.812, K50.813, K50.814, K50.818, K50.819, K50.90, K50.911, K50.912, K50.913, K50.914, K50.918, K50.919 |
| Ischemic colitis^†^ | ICD-10: K55.031, K55.032, K55.039, K55.042, K55.049 |

*International Statistical Classification of Diseases and Related Health Problems (ICD), 10^th^ revision

**Current Procedural Terminology

^†^A single encounter of any type with the relevant ICD-10 code qualifies as having the disease

*Manual chart review was performed to confirm the diverticular disease phenotypes, with 75-95% concordance for each phenotype.*
